# The Structure of Amyloid Versus the Structure of Globular Proteins

**DOI:** 10.3390/ijms21134683

**Published:** 2020-06-30

**Authors:** Piotr Fabian, Mateusz Banach, Katarzyna Stapor, Leszek Konieczny, Magdalena Ptak-Kaczor, Irena Roterman

**Affiliations:** 1 Institute of Computer Science, Silesian University of Technology, Akademicka 16, 44-100 Gliwice, Poland; piotr.fabian@polsl.pl (P.F.); katarzyna.stapor@polsl.pl (K.S.); 2 Department of Bioinformatics and Telemedicine, Jagiellonian University – Medical College, Lazarza 16, 31-533 Kraków, Poland; mateusz.banach@uj.edu.pl (M.B.); magdalena.ptak@uj.edu.pl (M.P.-K.); 3 Chair of Medical Biochemistry – Jagiellonian University – Medical College, Kopernika 7, 31-034 Kraków, Poland; mbkoniec@cyf-kr.edu.pl

**Keywords:** amyloids, hydrophobic core, synergy, external force field, entropy

## Abstract

The issue of changing the structure of globular proteins into an amyloid form is in the focus of researchers' attention. Numerous experimental studies are carried out, and mathematical models to define the essence of amyloid transformation are sought. The present work focuses on the issue of the hydrophobic core structure in amyloids. The form of ordering the hydrophobic core in globular proteins is described by a 3D Gaussian distribution analog to the distribution of hydrophobicity in a spherical micelle. Amyloid fibril is a ribbon-like micelle made up of numerous individual chains, each representing a flat structure. The distribution of hydrophobicity within a single chain included in the fibril describes the 2D Gaussian distribution. Such a description expresses the location of polar residues on a circle with a center with a high level of hydrophobicity. The presence of this type of order in the amyloid forms available in Preotin Data Bank (PDB) (both in proto- and superfibrils) is demonstrated in the present work. In this system, it can be assumed that the amyloid transformation is a chain transition from 3D Gauss ordering to 2D Gauss ordering. This means changing the globular structure to a ribbon-like structure. This observation can provide a simple mathematical model for simulating the amyloid transformation of proteins.

## 1. Introduction

Issues related to the mechanism of protein folding have gained a completely new perspective at the time of the appearance of amyloid proteins. The atypical structural form, unheard of among previously recognized proteins, makes this group particularly important. In addition, treating them as misfolded proteins in the context of pathological phenomena attracts the attention of many researchers [1]. The specificity of amyloid structures consists in generating aggregates with extruded fibrillar forms known as one-dimensional [2,3]. Achievement of the form with the possibility of unlimited growth of fibril is obtained due to the presence of β-structure, while helical forms do not create conditions for unlimited propagation [4]. The participation of charge–charge interactions is also emphasized in the process of amyloid transformation [5]. Generally, propagation of β-sheet systems is a fundamental factor favoring the formation of larger aggregates, but the role of facing edges of strands, especially from the point of view of the distribution of hydrophobicity, turns out to be critical [6]. Partial unfolding showing cooperativity is expected in the process of amyloid transformation [7,8]. The importance of the polar/hydrophobic ratio is treated as the main determinant for self-assembly [9,10]. The role of hydrophobicity is revealed by the introduced mutations affecting the ease of amyloid transformation [11,12]. Factors conducive to oligomerization are also identified as not necessarily buried [13]. However, hydrophobicity exposure is treated as a factor conducive to complexation [8,10,14].

The subject of analysis in the present work are amyloid proteins, the spatial structure of which is available in Protein Data Bank (PDB) [15]. Analysis of these proteins will be carried out on the basis of hydrophobic core assessment. Globular proteins represent a state stabilized by the presence of a hydrophobic core represented by a 3D Gaussian distribution [16,17]. The centric concentration of hydrophobicity together with its decreasing level as it moves away from the center of the molecule seems to be consistent with the form and properties of a 3D Gaussian distribution [16,17].

The actual distribution observed in globular proteins deviates more or less from such idealized distribution. However, the identification of local discrepancies is easy when using the description using the 3D Gaussian distribution (fuzzy oil drop model—FOD) [18].

Fibrillar structural forms built of numerous chains (proto- and superfibrils) show the presence of bands with a changing level of hydrophobicity with a high degree of incompatibility with the expected distribution expressed by the 3D Gaussian distribution [19]. However, it appears that the propagating fibril has a band with high hydrophobicity in the center of the fibril (including superfibril), which acts as a hydrophobic core in a linear form—a centrally located band surrounded by bands with lower hydrophobicity. In this situation, the distribution of hydrophobicity within a single chain with a flat structure can be expressed using a 2D Gaussian distribution. When such a distribution is used, a low level of hydrophobicity is observed on the outer part, while the central part expresses high hydrophobicity values [19].

The flat structure consistent with the 2D Gaussian distribution centralizes high hydrophobicity in the center of the ellipse, while exposing the two side surfaces to the environment. Such high hydrophobicity in the central part is not favorable from the point of view of interaction with the aquatic environment. Therefore, complexing the second and subsequent chains with similar characteristics reduces the surface of adverse contact with surrounding water, however, leaving the outer surfaces of the final peptides in this disadvantage. Complexing the next chain is therefore repeated in an endless process leading to the formation of linear fibrils of very long length.

The present work demonstrates the possibility of describing the distribution of hydrophobicity in single chains, the components of fibrils in the amyloid (both in proto- and superfibrillar forms), using 2D Gaussian distribution.

The description of the hydrophobicity distribution using the 3D Gaussian function distribution for super- and protofibrils and the 2D Gaussian function for single chains is presented in the present work, which is a continuation of the materials presented in [19]. The hypothesis proposed there was positively verified. Reference should also be made to the graphic presentation contained in [19], where T, idealized (3D or 2D Gauss function), and O, observed (the effect of inter-residual interaction), hydrophobicity profiles of all amyloids discussed here are shown.

The analysis was carried out for the amyloid forms available in the PDB database [20,21,22,23,24,25,26].

The main model applied for structure description is a fuzzy oil drop [17]. Only elements of the model that help interpret the final results are listed here.

The main assumption of the model is to adopt the possibility of describing the distribution of hydrophobicity in the globular protein molecule using a 3D Gaussian distribution. This is related to the commonly accepted model of stabilizing tertiary structure using the hydrophobic core present in proteins. The highest concentration of hydrophobicity is most often located in the center of the globular molecule with simultaneous exposure of hydrophilic residues (polar residues) on the surface, where the hydrophobicity is necessarily the lowest (on scale of 0–1, the hydrophobicity on the surface of the protein is zero). This distribution is defined as idealized, or theoretical—T. In this situation, the use of a 3D Gaussian distribution to describe the distribution of hydrophobicity in a protein seems obvious.

In fact, however, this distribution does not necessarily reflect such an idealized state. The actual distribution results from the distribution of residues with differentiated intrinsic hydrophobicity and from the distance between them on which the amount of hydrophobic interaction, Oi, depends. Levitt's function is used to describe hydrophobic interactions [27].

The idealized status of Ti is determined for the position of the so-called effective atom (the average position of atoms contained in a given amino acid). The same position also brings together the local interaction of a given residue with neighbors in the form of Oi.

After normalizing both these distributions, it becomes possible to compare them to determine the differences between them. For this purpose, Kullback–Leibler divergence entropy was used [28].

The assessment of the distribution of O against the reference distribution is broadened by introducing a second reference distribution R, which assumes even distribution of hydrophobicity throughout the molecule (Ri = 1 / N where N is the number of residues in the amino acid chain). The relative distance between O and T versus the relative distance between O and R allows to assess the degree of "closeness" to the O distribution in relation to two reference ones. Thus, RD (relative distance), the relationship of O–T to the sum of O–T and O–R distributions assuming a value of < 0.5, reveals the similarity of the O distribution to the T distribution and, thus, the presence of a central hydrophobic core [29].

The notation T–O–R means the distance of the distribution O against two references, T and R.

The use of the discussed model also enables the analysis of the status of chain fragments and the identification of those residues that significantly disturb the compatibility of the T and O distribution locally.

Determining the distribution of T and O after elimination of calculations of residues affecting the high value of RD lowers the value of RD. After reaching values lower than 0.5 it becomes possible to show the components of the hydrophobic core, i.e., those residues that are predicted to occupy the correct position in the protein body of the protein molecule.

This analysis describes the status of the globular protein.

However, if the structure is limited to two dimensions (flat structure), it becomes possible to use a 2D Gaussian distribution to describe the structure of the hydrophobic core in a 2D system. The compatibility of the O distribution with the T distribution in this case means the presence of a centric concentration of hydrophobicity, which is surrounded by a polar mantle only on a circle (ellipse). The central part – especially the one with high hydrophobicity values – remains open to interaction with the environment. Avoiding contact of the hydrophobic surface with water is the basis for spontaneously forming spherical micelles by bipolar molecules, and these are amino acids. Here, it is possible to achieve the state of a spherical micelle completely covered with polar groups on the surface, which guarantees an entropy beneficial system of protein–water relations. The ordering of flat types leads to the generation of the structure of the ribbon-like micelle. Exposure of hydrophobic residues in the central part of the flat hydrophobic core is a factor conducive to the generation of the ribbon-like structure, because complexing the second and subsequent molecules with a similar structure reduces the contact surface (it eliminates the surface that has just come into contact with the neighbor), but still leaves the surface of the initial molecules exposed to the environment and the end in the current fibril. This situation creates ideal conditions for the unlimited continued form of the ribbon-like micelle. This phenomenon is observed in the case of the formation of the ribbon-like micelle generated by bipolar molecules as well as in the forms of fibril in amyloids. 

For simplicity, the following notation is introduced: RD (3DG) means the RD value determined for the object in question using a 3D Gaussian distribution; RD (2DG) means the determined status of a given object while limiting the space in question to two dimensions (the component for the Z variable in the Gaussian distribution is eliminated) [19].

The postulated model for amyloid transformation is to orient the protein molecule in a coordinate system where the geometric center of the protein is located at (0,0,0). σX, σY, and σZ are determined for each axis. Then, structural changes in the changing external field in the form of a decrease in the σ value up to its disappearance (values close to zero) transform the globular form into a gradually flattened form to achieve a form described by the 2DG function.

Such a model is assumed for the representation of the in silico amyloid transformation process.

The amyloid proteins under consideration were subjected to the following analysis:

(1) orientation of the fibril in the coordinate system so that the geometric center of the fibril is located at the origin of the coordinate system
(2) orientation of the fibril, so that the position of the central chain in the fibril coincides with the XY plane and the Z axis is oriented according to the main axis of the fibril
(3) then the RD value was determined for the central chain (lying in the XY plane) treating this chain as part of the whole fibril ellipsoid
(4) for such orientation, RD (against 3D Gaussian distribution) was determined for the entire fibril, for the same orientation, RD (against 3D Gaussian distribution) was determined for the central chain treated as an individual structural unit for which the corresponding 3D G function was determined
(5) RD (2DG) was determined for the same orientation for the central chain (component for the Z variable is not present)
(6) for all determined RD values (points 3, 4, 5), the procedure of residue elimination was applied, which significantly influences exceeding the threshold of 0.5 for the RD value in order to identify residues showing the status as expected (distribution T).

The operation contained in point 6 helps to identify the degree of maladjustment by determining the number of residues that introduce local discordance. This is also done to identify the part of the chain that represents the O distribution in accordance with the T distribution. This, in turn, is interpreted as the determination of those parts of the chain that contribute to the structure of the hydrophobic core within the ribbon-like micelle which is amyloid fibril.

These calculations are designed to test the hypothesis that assumes that the status of a single chain, the fibril component, can be expressed using a 2D Gaussian distribution. The overarching goal is to be able to record the mathematical transformation of amyloids as a transition from ordering in accordance with a 3D Gaussian distribution to a 2D Gaussian form for the hydrophobicity distribution. 

## 2. Results

The 3D Gaussian distribution for calculating the value of the RD parameter was determined for the orientation of the entire aggregate, assuming the fibril axis is consistent with the Z axis. The point (0,0,0) coincides with the geometrical center of the chain located in a central position in the super- or protofibril. Under these conditions, this chain is oriented on the XY plane. In the case of the superfibril, the (0,0) point breaks with the center of the geometric chains that make up one common "floor".

To show the orientation of the 3D Gaussian distribution, the values of the parameters are also given: σX, σY, and σZ.

### 2.1. Superfibril Structure Analysis

The structure of the superfibrils described using the RD parameter shows values indicating significant mismatches of the hydrophobicity distribution O against the idealized distribution T (Table 1).

The σ parameter values express the proportions of the solid that the superfibril creates. In fact, the σZ value is much higher than the others. The low value of this parameter in the current analysis results from the small number of chains in the fibril structures available in PDB.

High values of the RD parameter result from the linear rather than the spherical distribution of hydrophobicity [19].

Is the superfibril formed according to the model based on contact of the exposed hydrophobic surface, as is observed in the case of protein complexes?

**Figure 1:**
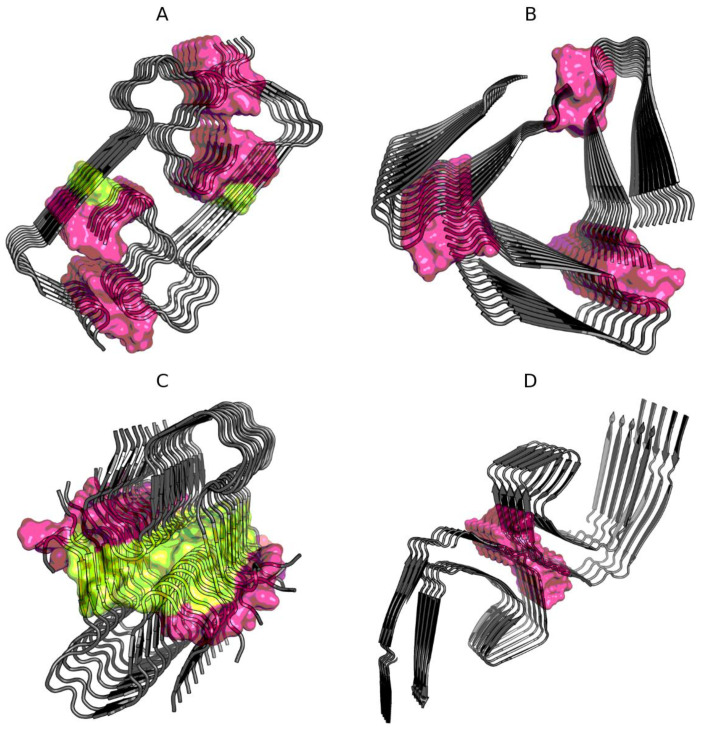
Location of interface residues in Aβ(1–40) (2MVX—**A**), Aβ(15–40) (2MPZ—**B**), Aβ(11–42)S (5KK3—**C**), and TAU (5O3L—**D**). Residues are distinguished according to Table 2: interface is shown as surface, with noncompliant residues in yellow (on **A** and **C**).

This question is answered by the results given in Table 2, where the status of residues remaining in contact between protofibrils is shown. Interfaces in Aβ(15–40) (2MPZ) and TAU (5O3L) show high compliance with the expected distribution. This means that the interaction, regardless of the level of hydrophobicity of the interface building residues, is part of the overall organization of the superfibril structure with an ordered distribution of hydrophobicity in accordance with the assumed model treating the complex as a spherical micelle. The interface structures in Aβ(11–42)S (5KK3) and Aβ(1–40) (2MVX) show an excess of the accepted cut-off level of 0.5. The reason lies in the incorrect status of the few interface members Figure 1.

The target subject of analysis is the status of individual chains, which in all amyloid structures available in PDB are flat. Considering the superfibril, the object of analysis is the system of chains that make up one layer of superfibril. For the analysis of the structure of such a layer, a description using a 3D Gaussian and a 2D Gaussian distribution was used, taking into account the two-dimensional form of such systems.

Individual treatment of a set of chains forming a common layer means that on these selected chains, a 3D Gaussian and a 2D Gaussian distribution are spread.

The results of such analysis are presented in Table 3 and Table 4, where the values of σ parameters determining the degree of approximation using a 2D Gaussian distribution are also given, and the dimension relative to the Z axis is not taken into account.

In addition, the status of these layers treated as a component of complete superfibril–3DG* was also shown. This means that a 3D Gaussian distribution is spread across the entire superfibril. However, the status of the selected layer is marked as the status of the component within the 3D Gaussian distribution spread over the entire superfibril.The need to eliminate a few residues in order to obtain a status of RD < 0.5, especially for amyloid Aβ, suggests quite good adjustment of the distribution of T and O within one layer treated as an individual unit and as a component of the superfibril.A different assessment applies to the layer in TAU, which, treated individually, shows a greater degree of maladjustment than as a component of the superfibril. This suggests that this form of protofibril exhibits a more stable state in the form of superfibril, of course, given only the status of the hydrophobicity distribution.

The number of residues introducing disorder in the meaning of the bell curve distribution given in the right column of Table 5 and Table 6 shows a degree of maladjustment to the idealized system comparable to other globular proteins. The number of these residues is comparable to that of globular proteins. 

On the other hand, comparing the status expressed using RD versus 3D Gaussian and 2D Gaussian distributions for the layer in these superfibrils reveals negligible differences.

### 2.2. Analysis of Protofibril Structure

The protofibril status described in Table 4 shows the presence of two protofibrils showing a system compatible with a 3D Gaussian distribution. This status is shown by 5KK3–Aβ (11–42)S and 2N0A–ASyn for segments 47–100.

The RD values determined for protofibril are higher compared to the superfibril status indicated as the preferred form of the superfibril, although the differences are very small. For Aβ(11–42)S and TAU, the protofibrillary form seems to be preferred.

Amyloid Aβ(11–42)S indicates a significantly preferred form of protofibril. The single-chain structure as well as the 3D structure of the entire Aβ(11–42)S protofibril appears to be very similar to the structure present in Aβ(11–42), which has the form of a protofibril.

In Table 4, the values of the σZ parameter are low relative to σX and σY. This is due to the low number of chains in the protofibril. In fact, fibrils contain a much higher number of chains. For such a structure, the σZ parameter has obviously higher values.

The minimal differences in RD values for 3D and 2D approximation result from the fact that the σZ parameters are low. Complete neglect of this parameter does not significantly affect the final result in the form of RD values.

**Figure 2:**
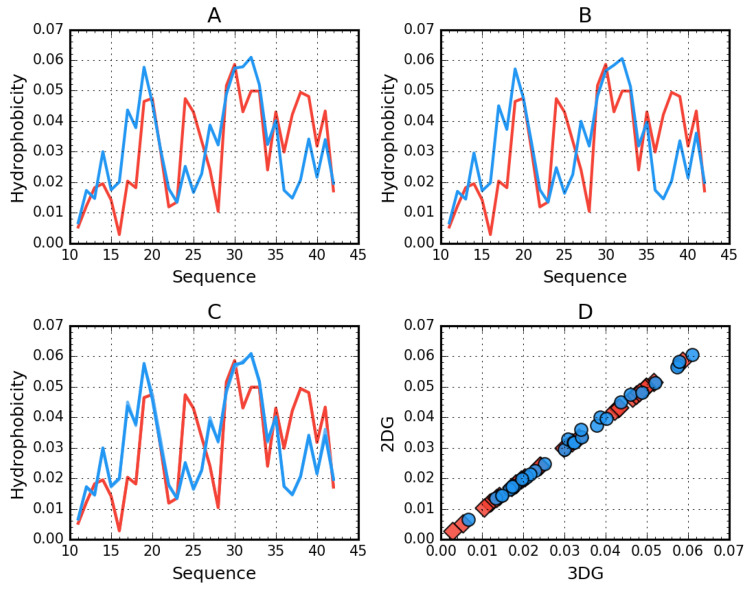
Theoretical (T, blue) and observed (O, red) hydrophobicity density profiles for Aβ(11–42) (2MXU). **A**—3DG. **B**—2DG. **C**—3DG (darker lines) vs. 2DG (lighter lines) T and O distribution comparison—line plot (differences are indistinguishable at this zoom level). **D**—3DG vs 2DG T (blue circles) and O (red rhombuses) distribution comparison—scatter plot.

**Figure 3:**
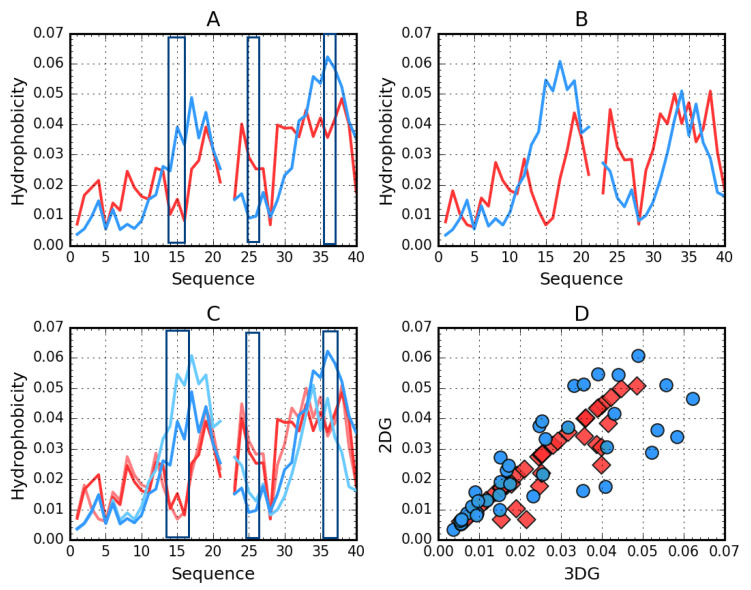
Theoretical (T, blue) and observed (O, red) hydrophobicity density profiles for Aβ(1–40) (2MVX). **A**—3DG. **B**—2DG. **C**—3DG (darker) vs. 2DG (lighter) T and O distribution comparison—line plot. **D**—3DG vs. 2DG T (blue circles) and O (red rhombuses) distribution comparison—scatter plot. The rectangles distinguish position of high discordance Ti versus Oi. The empty position in profiles due to the Osaka mutation E22Δ

Profiles presenting hydrophobicity distributions in all discussed amyloids were presented in [19]. For example, only two amyloids (Figure 2 and Figure 3) are shown here showing a status consistent and locally inconsistent with the idealized distribution.

Table 5 presents a set of RD parameters describing the status of individual chains in 3D Gauss and 2D Gauss representations. The results were obtained for calculations while determining the appropriate functions that were constructed for a single chain.

In addition, the status of a single chain as a component of protofibril is also shown. In this case, the generated function covers the entire protofibril as a structural unit.In this approach, it turns out that the single chain in the case of 2MXU Aβ(11–42) (Figure 2) and 2N0A ASyn for segment 47–100 shows the order as assumed. Other chains require elimination of residues introducing local disturbances to determine the part of the chain with the status consistent with the expected status. In some cases, the number of such residues is negligible. 5O3L (TAU) shows the highest degree of mismatch of the O distribution to the distribution. The large number of such residues in 5O3L (TAU) for the status of the chain as part of a larger structure expresses the opposite of the superfibrillary structure. This suggests that the superfibril form for this amyloid is more favorable in terms of the hydrophobic distribution.

Figure 2 visualizes the distribution of hydrophobicity in a single chain obtained for Aβ(11–42) (2MXU) in the 3DG and 2DG models. The differences are imperceptible, which is also expressed by very similar values of the respective RD parameters (Table 5). This amyloid is an example of an O distribution consistent with the T distribution. The hydrophobicity distribution in Aβ(1–40) (Figure 3) for both approximation reveals similar irregularity.

The number of residues introducing the disorder is comparable to that of globular proteins (Table 6). This means that the system is not perfectly compatible but within the standards observed in proteins in general.

**Figure 4:**
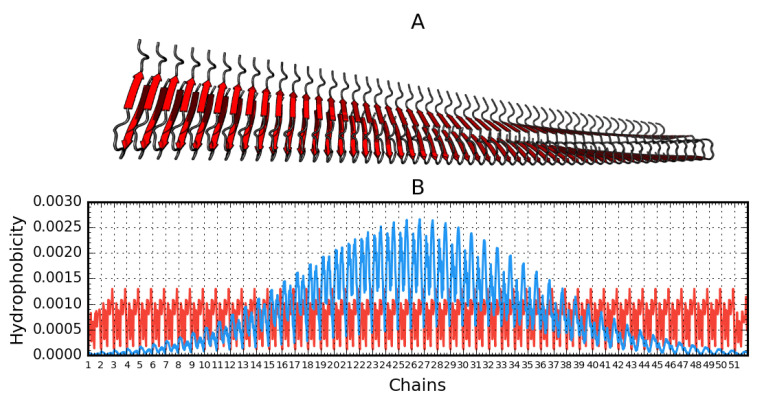
Aβ(15–40)_51_ (2MPZ*) structure generated based on operations that result from fibril symmetry. Contains 51 chains. **A**—3D presentation. **B**—theoretical (T, blue) and observed (O, red) hydrophobicity density distribution in this fibril.

The graphs in Figure 4 are intended to visualize the 3D form and its representation using T and O distributions. The linear nature of the fibril distributions becomes apparent relative to the expected distribution of soluble proteins expressing the globular state. The maximum location shift on the T distribution results from the spiral form of the fibril. This visualization aims to visualize the 3D Gaussian relationship for the globular form against the repetitive ordering in each unit (chain) ordering the distribution of the hydrophobicity of the component unit, a single chain. The paper shows 2D Gaussian type ordering for a single chain. The set of many chains generates a propagating band of high hydrophobicity in the central part of the fibril. 

The world of biologically active proteins is the world of globular proteins (at least if limited to soluble proteins). The distribution of hydrophobicity in the form of a 3D Gaussian distribution is obvious in this system. Protein folding in an aqueous environment, whose presence and impact on the folding process seems to be justified by taking into account the presence of an external field expressed by means of a 3D Gaussian distribution.

The flatness of this structure results from the optimal interaction of the monomers in the complex, resulting in a 3D Gaussian type distribution. The resulting flat structure is in some sense imposed by the target protein. 

In the amyloid, all "partners", individual chains, represent an equivalent state. There is no target molecule here. The structure is the result of synergy in which chains of identical sequence and identical structural form participate. There is no target here to adapt to and which would impose chain matching. Here, synergy is a system of equal partners, single chains.

Therefore, based on the results presented in the present work and postulated in [19], it is planned to fold polypeptide chains, components of amyloid in the presence of an external force field expressing the presence of the environment by means of a 2D Gaussian distribution. 

Simulations of the process of folding polypeptide chains, the components of amyloids discussed here, carried out in an environment the specificity of influence of which is expressed by a 3D Gaussian distribution, showed little possibility of generating globular structures [30,31,32,33,34].

All studies on in vitro amyloid formation indicate the need for external factors [35]. An example of such observation is also the production of amyloids in laboratory conditions by shaking. Both the chemical factors mentioned in [35] and the shaking process result not so much in a direct interaction with the polypeptide chain, but in a change in water structuring. Shaking increases the share of water structuralization at the water/air phases, the structuralization of which is definitely different from that seen in conditions without the presence of external factors [36,37,38]. An example can also be the participation of TFE (tri-fluoro-ethanol), the presence of which promotes the formation of amyloid. There is no doubt that such a relationship, as TFE significantly affects the structuring of water, which, despite numerous studies, remains unknown [39].

The use of 3D Gaussian and 2D Gaussian distributions for amyloids raises the question of the impact of the σZ parameter value on the function form. Assuming that the structure of a single chain in an amyloid is a structure with a hydrophobicity distribution ordered according to the 2D Gaussian distribution and is derived from the 3D Gaussian structure (e.g., Immunoglobulin G (IgG) domain V Light chain and its amyloid form), the transformation process can be expressed as a transformation of the 3D Gaussian form into the 2D Gaussian form, and thus, the value of one of the Sσ parameters decreases (in our example, it is σZ) to values close to zero. In contrast, the formation of fibril is the process of increasing σZ theoretically to infinity. 

For the σZ parameter going to 0, the 3D Gaussian function takes a shape similar to the Dirac delta function. For the σZ parameter going to infinity, the 3D Gaussian function takes values close to 0. However, if we apply it to the physical model, it can be assumed that in this situation, the function for the component dependent on the variable Z takes a constant value.

Therefore, it is assumed that the given set of two functions and the process of their mathematical transformation can act as a model for the process of amyloid transformation.

Calculations of protein spatial structures in the environment represented by 2D Gaussian distribution are planned. Simulation of the folding process in the environment expressed by the 3D Gaussian distribution was made for amyloid chains without (or negligible) globular forms [30,31,32,33].

T and O profiles of all 3DG versions of amyloids discussed here are available in [19]. In the present work, only two selected profiles are shown with a high approximation of the representation using two forms of a Gaussian distribution avoiding the redundancy of the presented results.

## 3. Discussion

The hypothesis expressing the amyloid as the ribbon-like structural form given in [18] was proven in the work presented here. The hydrophobicity distribution in globular proteins can be expressed by a 3D Gaussian function which well represents the presence of centric hydrophobic core. The amyloid structure appears to be highly discordant with this form of representation. The distribution of hydrophobicity in ribbon-like form, however, can be represented by a 2D Gaussian function. It appears that all amyloid structures available in PDB show flat forms for individual chains. This is why the 2D Gaussian representation may be accepted to express the status of individual chains in fibril. This is why σZ, which is close to zero for an individual chain, appears to increase σZ →∞ for elongated fibrils. The positive verification of this hypothesis allows the simulation of amyloid formation in silico as a folding process in the environment expressed by σZ → 0. The centric concentration of hydrophobicity in one slice assumes the polar groups on the circumference of a circle (ellipse). However, two surfaces of high hydrophobicity are still exposed. This exposition supports the elongation of the fibril. The simulations of polypeptide chain folding under influence of a 3D Gaussian function expressing the presence of an external force field (water environment) were applied for globular proteins. The folding simulation in the 2D Gaussian external force field is currently carried out by authors of this paper. 

**Figure 5:**
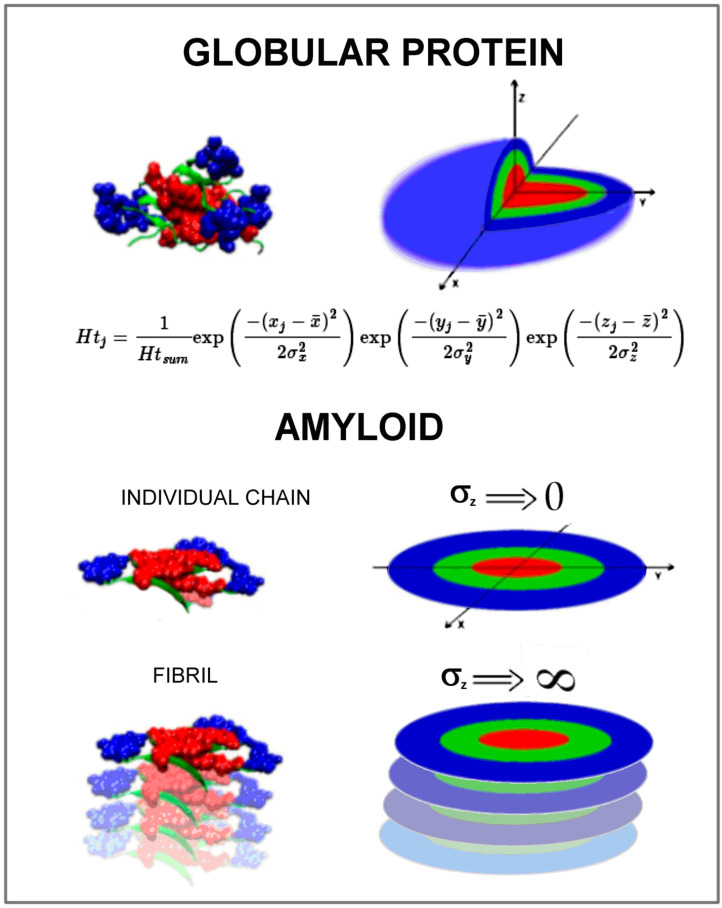
The visualization of hydrophobicity distribution expressed by a 3D Gaussian function in globular protein in comparison to the status appearing in an individual polypeptide chain in amyloid form expressed by the 2D Gaussian function. The manipulation of the σZ parameter seems to be critical for the simulation of amyloidosis transformation in silico.

The summary in graphic form is given in Figure 5. It represents the model assumed to simulate the amyloid transformation in silico.

The globular form of proteins usually represents the hydrophobicity distribution according to a 3D Gauss function. The local discrepancy is observed mainly in function-related areas. The amyloid form of individual chains is flat with hydrophobicity concentrated in the central part, however, expressed by a 2D Gaussian form. This model is planned to be applied for the simulation of amyloid transformation. This paper aimed to check the reliability of this hypothesis.

## 4. Materials and Methods

### Data

The proteins that are the subject of the analysis are given in Table 7. The amyloid structures available in PDB are summarized. The analysis was performed for single chains, protofibrils, and superfibrils.

The names of the proteins in question are also shown. Aβ(11–42)S is the superfibril form for this amyloid. 

The structure of the fibrillar form in 2N0A (ASyn) is represented by fragments 30–100 but also for segments 47–100, which form an unbranched order.

A fibril constructed in silico by chain duplication according to the protofibril scheme 2MPZ Aβ(15–40) was added to the list of amyloids.

## 5. Conclusions

The paper demonstrates the validity of expressing the ordering of the hydrophobicity distribution in the form of a 2D Gaussian distribution for single polypeptide chain structures present in amyloid fibrils in the analysis. This is due to the flat structural form of individual polypeptide chains in available forms of amyloids. Local inconsistencies, often limited to individual residues, represent a neglected level from the point of view of the entire structure. Similar residue numbers expressing a local mismatch with the idealized distribution are identified by globular proteins (Oi versus Ti comparison).

The simulation of the process of structural changes starting from the globular form with the influence of the environment expressed by means of a 3D Gaussian distribution by introducing elements of unfolding (in an appropriately adapted degree) and gradual change of the external field form to a 2D Gaussian distribution becomes possible. During the simulation, the shape of the ellipsoid in which the protein folds (outer field) takes different forms depending on the relation of the σ parameter values. Directing the folding process towards reducing the value of one of the σ parameters should lead to a flat form typical for amyloids (at least so far, when the number of recognized amyloid structures is limited).

The use of the proposed description also allows the determination of the polarity/nonpolarity ratio, which determines the specific need for the number of polar residues needed to cover the contact surface with water. In the case of the globular form, this proportion is expressed as R^3^, while in the case of the ribbon-like micelle form, the demand for polar residues is expressed as R^2^, where R is the radius of the hydrophobic part (radius of the hydrophobic core, which can also be expressed as a dependence on the approximate radius of the entire globular protein) [9].

## Figures and Tables

**Table 1:** Relative distance (RD) values for superfibrils. RD value calculated for the 3D Gaussian distribution covering the entire superfibril, the chains given in column 2. Chains in parentheses—middle chains in the fibril. The values of the parameters σX, σY, and σZ are given for expressing the aggregate orientation assuming compliance of the Z axis with the fibril axis. The chains given in brackets are oriented in the XY plane.

Protein	Chains	RD	σX	σY	σZ
Aβ(1-40) (2MVX) Aβ(15–40) (2MPZ) Aβ(11–42)S (5KK3) TAU (5O3L)	A-J (F) A-a (N) A-R (J) A-J (F)	0.603 0.613 0.571 0.626	12.393 15.496 13.797 25.939	14.480 15.039 12.635 13.827	8.6907 14.156 14.690 11.118

**Table 2:** RD values for interfaces in superfibrils. Residues present in the interface are given. The term Aβ(11–42)S means Aβ(11–42) amyloid in the form of a superfibril.

Protein	RD	Interface	Eliminated
Aβ(1–40) (2MVX) Aβ(15–40) (2MPZ) Aβ(11–42)S (5KK3) TAU (5O3L)	0.557 0.443 0.592 0.388	3,4,13,15,28–30,37–40 28,29,31,38,40 13,15,17,34–37 331–336,338	15 – 15,17,34,35 –

**Table 3:** Status of a set of chains forming a common "floor" within a superfibril expressed by means of a 3D Gaussian (3DG) distribution: a set of chains treated as an individual structural unit; 3D Gauss* (3DG*): determined for a set of chains treated as a superfibril component; 2D Gauss (2DG): a set of chains treated as an individual structural unit. RDe: RD values obtained after elimination of number of residues listed in the right column. The residues that are different for the set of eliminated residues are underlined.

Protein	Chains	RD 3DG 3DG * 2DG	RDe 3DG 3DG * 2DG	Number of Residues Eliminated to Reach RD Value Below 0.5
Aβ(1–40) (2MVX)	C, H	0.578 0.623 0.576	0.498 0.499 0.487	6 11 6
Aβ(15–40) (2MPZ)	N, O, P	0.630 0.584 0.644	0.498 0.497 0.481	3 5 4
Aβ(11–42)S (5KK3)	E, N	0.607 0.536 0.609	0.489 0.495 0.494	4 4 4
TAU (5O3L)	E, F	0.666 0.637 0.655	0.485 0.497 0.495	28 18 22

**Table 4:** RD values for protofibrils. RD value calculated for a 3D Gaussian distribution encapsulating the whole protofibril. The values of the parameters σX, σY, and σZ are given for expressing the aggregate orientation. Z axis oriented according to the long axis of the fibril. A set of chains included in the protofibrils is given. A central chain that occupies a position in the XY plane is given in parenthesis. Value provided as bold—status consistent with idealized distribution.

Protein	Chains	RD	σX	σY	σZ
Aβ(11–42) (2MXU)	A-L (F)	0.655	10.06	13.61	13.84
Aβ(1–40) (2MVX)	A, B, C, D, E (C)	0.619	13.87	12.42	8.34
Aβ(15–40) (2MPZ)	B, E, H, K, N, Q, T, W, Z	0.683	12.04	10.61	11.13
Aβ(11–42)S (5KK3)	A-I (E)	0.525	12.38	10.46	13.98
TAU (5O3L)	A, C, E, G, I (E)	0.604	14.65	15.42	8.28
ASyn (2N0A)	A- J (E)	0.576	18.94	18.7	14.14
	30–100	**0.486**	13.48	11.88	14.19
	47–100				
ImVA (6HUD)	A-E (C)	0.724	15.62	12.5	9.03
Aβ(15–40)_51_ (2MPZ)	51 chains	0.847	15.1	14.76	47.11

**Table 5:** Status of single, centrally located protofibril chains treated as an independent, individual structural unit (Gaussian distributions were determined for the mentioned chains). The RD and σ parameter values are given for 3D and 2D Gaussian distributions, respectively. Protofibril orientation as in Table 4.

Protein	Chain	RD	σX	σY	σZ
		3DG			
		2DG			
Aβ(11–42) (2MXU)	F	0.441	9.289	11.829	4.625 –
		0.445	9.289	11.829	
Aβ(1–40) (2MVX)	C	0.609	13.639	12.227	4.961
		0.603	13.639	12.227	–
Aβ(15–40) (2MPZ)	N	0.667	11.195	7.515	4.921
		0.669	11.195	7.515	–
Aβ(11–42)S (5KK3)	E	0.547	9.912	9.972	4.664
		0.548	9.912	9.972	–
TAU (5O3L)	E	0.602	14.304	15.102	5.121
		0.605	14.304	15.102	–
Asyn (2N0A)	E (30–100)	0.548	16.52	17.019	5.165
		0.551	16.52	17.019	–
	E (47–100)	**0.375**	13.092	11.57	4.703
		**0.378**	13.092	11.57	–
ImVA (6HUD)	C	0.72	15.517	12.098	5.768
		0.729	15.517	12.098	–

**Table 6:** Status of a single chain expressing differences between the description using 3D Gauss (3DG) and 2D Gauss (2DG) models. 3DG—for the structure of an individual chain in the 3D Gauss representation, 3DG*—RD values for determining the status of the chain treated as a component of the entire protofibril, 2DG—status for the 2D Gauss representation. RDe—RD values obtained after eliminating the residues listed in the right column. The residues that are different for the two compared forms of description are highlighted.

ASyn (2N0A) 30–1000.527	0.487	9	
0.441			
0.551	0.484	9	
Aβ(15–40)_51_ (2MPZ*)	0.505	0.479	38

**Table 7:** List of amyloids analyzed in the present work. The composition of superfibrils, if applicable, and the number of protofibrils in superfibrils, if applicable, (number in parentheses) are given. The number of "floors" (layers) in the super- and protofibril construction is also given. 2MPZ* denotes Aβ(15–40)_51_ D23N, a 51-layer protofibril generated in silico according to the 2MPZ protofibril scheme.

PDB ID	Name	Structure	Number of layers	Ref
2MXU	Aβ(11–42)	Protofibril	12	[20]
2MVX	Aβ(1–40) E22Δ	Superfibril (2)	5	[21]
2MPZ	Aβ(15–40)D23N	Superfibril (3)	9	[22]
5KK3	Aβ(11–42)S	Superfibril (2)	9	[23]
5O3L	TAU	Superfibril (2)	5	[24]
2N0A	ASyn	Protofibril	10	[25]
	30–100	30–100	10	
	47–100	47–100	10	
6HUD	IgG V domain ImVA	Protofibril	5	
2MPZ*	Aβ(15–40)_51_ D23N*	Protofibril	51	[26]

## Abbreviations

ImVA	IgG domain V
